# Assessment of the efficacy of firocoxib (Previcox®) and grapiprant (Galliprant®) in an induced model of acute arthritis in dogs

**DOI:** 10.1186/s12917-019-2052-0

**Published:** 2019-08-29

**Authors:** Andrea García de Salazar Alcalá, Lucile Gioda, Alia Dehman, Frederic Beugnet

**Affiliations:** 1 Avogadro LS, Parc de Génibrat, 31470 Fontenilles, France; 2 Hyphen-stat, 195, route d’Espagne BP13669, 31036 Toulouse Cedex 1, France; 3 Boehringer Ingelheim Animal Health, 29, avenue Tony Garnier, 69007 Lyon, France

**Keywords:** Dog, Firocoxib, Grapiprant, Lameness ratio, Force-plate, Osteoarthritis

## Abstract

**Background:**

Non-steroidal anti-inflammatory drugs (NSAIDs) are an important tool in the management of canine osteoarthritis, with the most recent introduction into the category being grapiprant, a piprant that selectively targets the EP4 prostaglandin receptor. To date there have been no efficacy studies comparing grapiprant with other NSAIDs. A randomized, two-sequence, assessor-blinded study involving two separate experiments was undertaken to measure the potency and persistence of acute pain control over 24 h resulting from a single oral dose of either firocoxib (Previcox®) or grapiprant (Galliprant®) in an acute arthritis model.

**Results:**

Force-plate derived lameness ratios (0, no force recorded on the plate; 1, normal force) for the untreated group remained at 0 for most post-arthritis induction (PAI) assessments in both experiments. Throughout Experiment 1, mean PAI lameness ratios of the firocoxib-treated group remained at or above 0.80. In the grapiprant-treated group, ratios were 0 at 5 and 7 h PAI (7 and 9 h post-treatment), and 0.16 at 10 h PAI (12 h post-treatment). For lameness ratios, relative to the firocoxib group, the control and grapiprant group ratios were significantly lower at each PAI assessment (*p* ≤ 0.026 and *p* < 0.001, respectively), except at 1.5 h PAI at which acute pain was still not installed in untreated control dogs. In Experiment 2 the mean lameness ratios for the control group were 0 at 3, 5 and 7 h PAI, and in the grapiprant group at 5, 7 and 10 h PAI (i.e., 19, 21, and 24 h post-treatment). In the firocoxib group the lowest mean lameness ratio of 0.36 occurred at 3 h PAI (i.e. 17 h post-treatment). Except at 1.5 and 3 h PAI (i.e. 15.5 and 17 h post-treatment), due to the needed time for pain to install in the untreated control dogs, the lameness ratio differences between the firocoxib and both the control and grapiprant groups were significant at all assessments (*p* ≤ 0.033 for both groups). No significant differences were detected between the grapiprant and control groups in either experiment.

**Conclusions:**

Firocoxib treatment prior to induction of arthritis in dogs resulted in a high level of analgesia from the first post-treatment assessment at 1.5 h through 24 h post-treatment. The reduction in lameness provided by firocoxib was consistently superior to that provided by grapiprant, which was not significantly different from untreated controls.

## Background

The use of non-steroidal anti-inflammatory drugs (NSAIDs) is an important tool in the management of canine osteoarthritis. The beneficial anti-inflammatory effects of NSAIDs arise from inhibition of the cyclooxygenase enzymes (COX-1 and COX-2) that are produced by the breakdown of arachidonic acid resulting from cell-wall damage [1]. COX-1 is recognized as constitutive with importance in maintaining homeostasis through normal prostaglandin-related physiologic functions. The belief that the inducible COX-2 was primarily responsible for pathologic processes led to the development of NSAIDs, collectively coxibs, that would selectively target COX-2 while sparing COX-1 [2]. Members of the coxib class that are now available for the treatment of canine osteoarthritis include cimicoxib, deracoxib, firocoxib, mavacoxib and robenacoxib. Thus, through sparing of COX-1, the objective of these coxibs is to reduce the risk of adverse effects while maintaining the full benefit of anti-inflammatory and analgesic actions that would alleviate the inflammation and pain due to osteoarthritis. Recently, recognition that COX-2 also has constitutive activity led to a search for drugs with modes of action, other than cyclooxygenase inhibition, to reduce inflammatory pathways [3].

A recent outcome of that research has been the release of the piprant molecule, grapiprant, which selectively targets the EP4 prostaglandin receptor without inhibiting COX [3]. It is believed that this mechanism of action would not interfere with the production of many of the prostanoids that cascade from the COX pathways affected by traditional NSAIDs. Nevertheless, to date there is no clinical evidence of any safety advantages over other NSAIDs and there have been no reports of efficacy comparisons of grapiprant with other NSAIDs.

To investigate the comparative efficacy of grapiprant, a randomized, two-sequence, assessor-blinded study was designed to measure the potency and persistence of pain control over 24 h resulting from a single oral dose of either firocoxib (Previcox®) or grapiprant (Galliprant®) in an acute arthritis model [4–8]. The acute arthritis model, based on the intra-articular injection of urate crystals, induces pain in one joint for duration of around 10 h, with a peak of pain between 3 to 7 h post-injection [5, 7, 8]. The exact same methodology was already used in 2017 to compare the pain control of firocoxib and robenacoxib [5]. The study consisted of two experiments performed over two different periods following administration of the drugs. In the first experiment, arthritis was induced approximately two hours post treatment, allowing assessing pain control from 3.5 h to 12 h post-treatment (assessment times at 1.5, 3, 5, 7, and 10 h post induction). In the second experiment, acute arthritis was induced 14 h post-treatment, allowing to assess pain control until 24 h post-treatment. As these drugs are intended to be administered daily (i.e., every 24 h), such design including two experiments allowed to assess the maintenance of pain control over an entire day.

## Results

The results present the pain control in dogs after induction of a transient joint arthritis (see Methods). The acute pain was obtained after intra-articular injection of 1 mL of a sodium urate crystal suspension into the femorotibial joint using a 30 mm by 1 mm sterile needle (Experiment 1 – right joint; Experiment 2 – left) in anaesthetized dogs [5]. All dogs were fully awake by the time of the start of the assessments (beginning 1.5 h after anaesthesia).

In Experiment 1, the force plate and video recordings were available for 5 of the 6 dogs in the untreated control group because one dog did not show any sign of lameness or pain at any time point, indicating that arthritis induction had failed, and so data from that control dog were not included in lameness calculations (i.e. 5 control dogs, 6 fipronil treated and 6 grapiprant treated dogs included in Experiment 1). Arthritis induction was successful in this control dog during the Experiment 2.

In experiment 1, for one firocoxib treated dog, the values were missing at 7 h post arthritis induction (PAI) because of a computer error; therefore the statistical analysis included 5 fipronil treated dogs at 7 h PAI, and 6 at the other time-points.

In Experiment 2 a computer error in the force plate and video recordings for one dog in the firocoxib group at the final time point assessment (10 h PAI, i.e. 24 h post-treatment) prevented inclusion of data from that dog at that particular time-point.

Other than for the induced lameness, no abnormal clinical signs were recorded during the study in any group.

### Lameness (vertical force) ratios

**Fig. 1:**
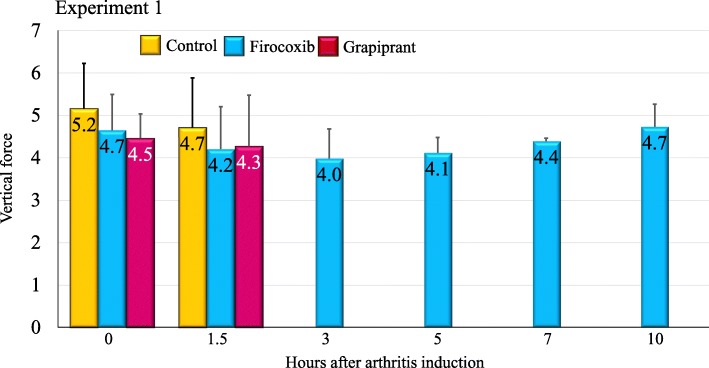
Group mean vertical force values for untreated control dogs or dogs treated with either firocoxib or grapiprant two hours prior to induction of arthritis (Experiment 1)

**Fig. 2:**
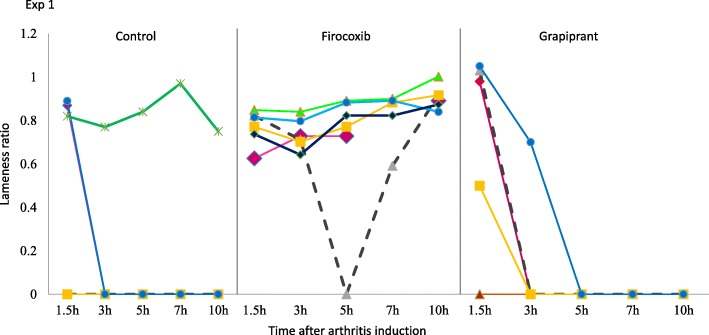
Individual lameness ratios for untreated control dogs or dogs treated with either firocoxib or grapiprant two hours prior to induction of arthritis (Experiment 1)

**Fig. 3:**
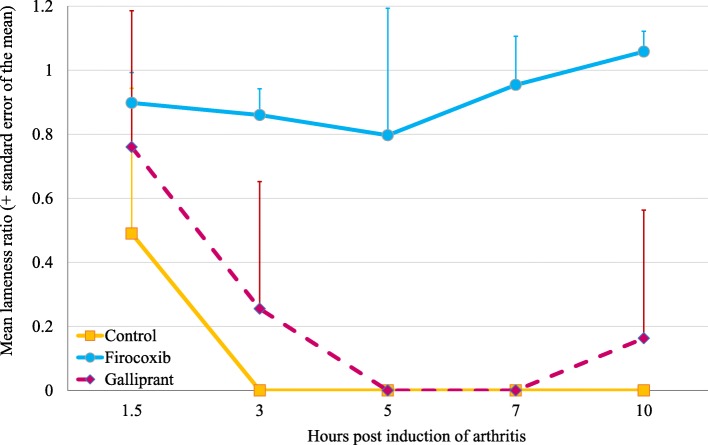
Group mean lameness ratios for untreated control dogs or dogs treated with either firocoxib or grapiprant two hours prior to induction of arthritis (Experiment 1)

In Experiment 1, prior to arthritis induction all dogs in the study had mean vertical force measurements (the mean of three crossings of the force plate) of at least 3.5 kg, with means in the control, firocoxib and grapiprant groups of 5.2, 4.7 and 4.5 kg, respectively, with no significant difference between groups (Fig. 1). These vertical force measurements before the induction of arthritis provided baseline data for calculation of individual lameness ratios for each dog at each time-point PAI (i.e. 1.5, 3, 5, 7 and 10 h PAI). The assessment times were based on available published data regarding the model, from appearance to peak and decrease of pain within 10 h [5, 7, 8]. A lameness ratio of 0 indicates that, due to acute pain, no pressure was applied from the affected limb onto the force plate, while a ratio of 1 indicates that the force applied to the plate was the same as that applied prior to arthritis induction. The mean lameness ratios for all groups had values > 0 at 1.5 h PAI indicating that pain was still not installed in the untreated control dogs. Lameness ratios for the control group remained at 0 for all subsequent assessments, indicating that acute pain was assessed at first 3 h PAI in control dogs (Table 1, Fig. 2). In the firocoxib-treated group the mean lameness ratio remained at or above 0.80 throughout the experiment (Table 1, Fig. 3). In the grapiprant treated group, ratios were 0 at 5 h and 7 h PAI (i.e., at 7 and 9 h post-treatment), and were 0.16 at 10 h PAI (i.e. 12 h post treatment). One of the firocoxib-treated dogs was non-weight-bearing at a single point (5 h PAI). Four of the six grapiprant-treated dogs were non-weight-bearing in the induced limb from 3 h, and one from 1.5 h PAI, to the end of the experiment. Relative to the firocoxib group, except at 1.5 h PAI related to the low pain level in untreated dogs, lameness ratios in the control group were significantly lower at each PAI assessment (*p* ≤ 0.026). Lameness ratios in the grapiprant group were significantly lower than in the firocoxib group at all assessments (*p* < 0.001) except at 1.5 h PAI (Table 1). No significant differences were observed between the grapiprant and the control groups.

**Table 1:** Group mean (minimum-maximum) lameness ratios in untreated control dogs, and in dogs treated with either firocoxib or grapiprant

Time (hours post induction)	Control	Firocoxib	Grapriprant
Experiment 1 - treatments administered 2 h prior to induction of arthritis			
1.5	0.49 (0.00–0.89)^a^	0.90 (0.73–0.99)	0.76 (0.00–1.05)^b^
3	0.00 (0.00–0.00)^c^	0.86 (0.75–0.98)^c^	0.26 (0.00–0.83)^d^
5	0.00 (0.00–0.00)^c^	0.80 (0.00–1.04)^c^	0.00 (0.00–0.00)^d^
7	0.00 (0.00–0.00)^c^	0.95 (0.69–1.05)^c^	0.00 (0.00–0.00)^d^
10	0.00 (0.00–0.00)^c^	1.06 (0.98–1.17)^c^	0.16 (0.00–0.98)^d^
Experiment 2 - treatments administered 14 h prior to induction of arthritis			
1.5	0.28 (0.00–0.66)^a^	0.75 (0.00–0.97)	0.61 (0.00–1.02)^b^
3	0.00 (0.00–0.00)	0.36 (0.00–0.84)	0.00 (0.00–0.65)
5	0.00^d^ (0.00–0.00)^d^	0.45 (0.00–0.99)^d^	0.00 (0.00–0.00)^e^
7	0.00 (0.00–0.00)^c^	0.80 (0.00–1.07)^c^	0.00 (0.00–0.00)^d^
10	0.35 (0.00–0.87)^f^	0.91 (0.66–1.05)^f^	0.00 (0.00–0.00)^g^

Different superscripts in the same row indicate statistically significant differences: a,b *p* = 0.026; c,d *p* < 0.001; e,d *p* = 0.033; f,g *p* ≤ 0.006

**Fig. 4:**
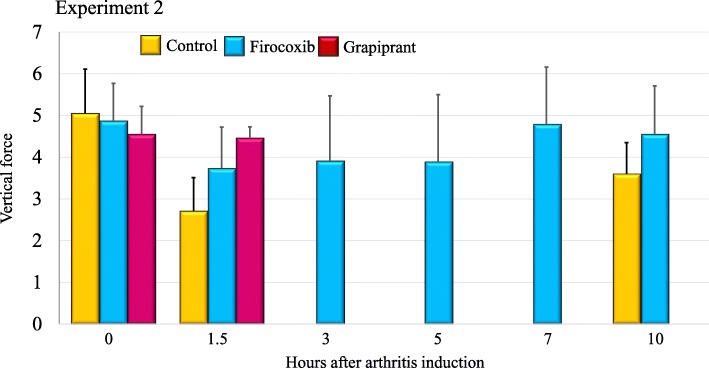
Group mean vertical force values for untreated control dogs or dogs treated with either firocoxib or grapiprant two hours prior to induction of arthritis (Experiment 2)

**Fig. 5:**
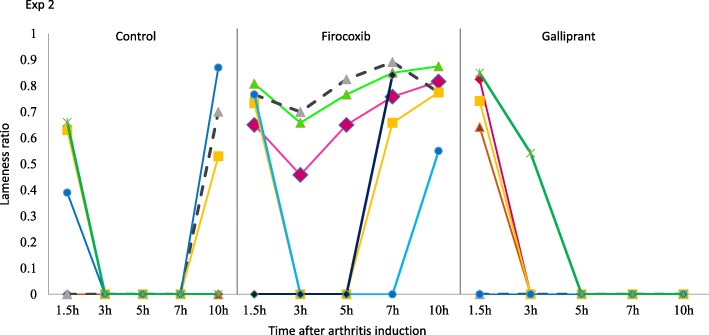
Individual lameness ratios for untreated control dogs or dogs treated with either firocoxib or grapiprant two hours prior to induction of arthritis (Experiment 2)

**Fig. 6:**
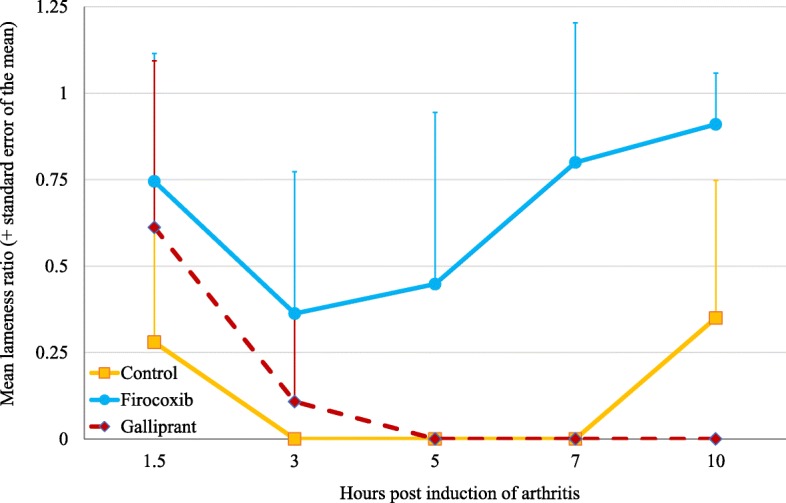
Group mean lameness ratios for untreated control dogs or dogs treated with either firocoxib or grapiprant two hours prior to induction of arthritis (Experiment 2)

In Experiment 2, prior to arthritis induction vertical force measurements for all dogs were at least 3.8 kg, with means in the control, firocoxib and grapiprant groups of 5.1, 4.9 and 4.6 kg, respectively, with no significant differences between the groups (Fig. 4). The mean lameness ratios for the control group were 0 at 3, 5 and 7 h PAI (i.e., 17, 19 and 21 h post-treatment), and in the grapiprant group at 5, 7 and 10 h PAI (i.e., at 19, 21 and 24 h post-treatment) (Table 1, Fig. 5). In the firocoxib group the lowest mean lameness ratio of 0.36 occurred at 3 h PAI. Three of the six control dogs were non-weight-bearing from 1.5 h or 3 h to the end of Experiment 2, and all dogs in this group were non-weight-bearing at 3, 5 and 7 h PAI (Fig. 6). Three of the six firocoxib-treated dogs were non-weight-bearing at 3 and 5 h PAI, one of these dogs was also non-weight-bearing at 7 h, and another one was also non-weight-bearing at 1.5 h. Five of the six grapiprant-treated dogs were non-weight-bearing in the induced limb from 3 h and one from 5 h to the end of Experiment 2. For the lameness ratio comparisons, except at 1.5 h PAI (i.e., 15.5 h post-treatment administration) the differences between the firocoxib and the control group were significant at all assessment time-points (*p* ≤ 0.033). Relative to dogs treated with firocoxib, except at 1.5 h PAI, the lameness ratios observed in the grapiprant-treated dogs were significantly lower at all assessments (*p* ≤ 0.033) (Table 1). No significant differences were detected between the grapiprant and control groups.

### Visual lameness scores

Visual lameness scores, corresponding to a blinded operator observation, ranging from 0 to 5, were used as secondary criteria of assessment. In both experiments, prior to arthritis induction the combined lameness score (sum of standing and walking scores) for all dogs was zero, indicating no sign of lameness. In Experiment 1, all dogs in the control group had maximum lameness scores (score of 5) from 3 h PAI (i.e., 5 h post-treatment) to the last time point (10 h PAI, i.e. 12 h post treatment) (Table 2). In the firocoxib group, a lameness score was positive in one dog at 1.5 h PAI (score 2) and two dogs at 3 h (scores of 1 and 3). At some time-points during this experiment, every dog in the grapiprant group was given a lameness score of 5. Over the entire experiment, mean scores for the firocoxib group were significantly lower than the mean scores of the control (*p* < 0.001) and grapiprant groups (*p* < 0.001) with no significant difference between the grapiprant and control groups (*p* = 0.204).

**Table 2:** Group mean (minimum – maximum) visual lameness scores based on summation of standing and walking lameness scores in untreated control dogs, or in dogs treated with either firocoxib or grapiprant

Time (hours post induction)	Control	Firocoxib	Grapiprant
Experiment 1 - treatments administered 2 h prior to induction of arthritis			
0 (pre-arthritis induction)	0 (0–0)	0 (0–0)	0 (0–0)
1.5	2 (0–5)	0 (0–2)	2 (0–5)
3	5 (5–5)	0 (0–3)	4 (1–5)
5	5 (5–5)	0 (0–0)	5 (5–5)
7	5 (5–5)	0 (0–0)	5 (4–5)
10	5 (5–5)	0 (0–0)	4 (1–5)
Experiment 2 - treatments administered 14 h prior to induction of arthritis			
0 (pre-arthritis induction)	0 (0–0)	0 (0–0)	0 (0–0)
1.5	5 (4–5)	2 (0–5)	2 (0–5)
3	5 (5–5)	3 (0–5)	5 (4–5)
5	5 (5–5)	4 (0–5)	5 (5–5)
7	5 (5–5)	1 (0–5)	5 (5–5)
10	4 (0–5)	0 (0–1)	5 (5–5)

In Experiment 2, the combined visual lameness score was at the maximum for all dogs in the control group at 3, 5 and 7 h PAI (Table 2). There was more evidence of lameness in the firocoxib treated dogs in this experiment than in Experiment 1, with all dogs scoring the maximum lameness value of 5 at 3, 5 and 7 h PAI (i.e. 17, 19 and 21 h post treatment). In the grapiprant group, all dogs scored the maximum lameness value of 5 at 3, 5, 7 and 10 h PAI (i.e. 17, 19, 21, and 24 h post treatment). Over the entire experiment, mean scores of the firocoxib group were significantly lower than the mean scores of the control (*p* < 0.001) and grapiprant groups (*p* < 0.001), with no significant difference between the grapiprant and the control groups (*p* = 0.471).

## Discussion

Force plate studies have been described as the gold standard for assessing improvements in lameness following surgical stabilization of cranial cruciate ligament–deficient stifle joints and for assessing lameness in osteoarthritic dogs [4–6]. These studies have also become an established means of evaluating anti-inflammatory and analgesic drugs such as NSAIDs [4–11]. In each group in the study reported herein, the results of the initial (T0) force plate measurements in each group in both experiments indicate the consistency of the force plate methodology, while the technique of inducing lameness was successful in all but one dog in the untreated control group in Experiment 1 (Fig. 1).

The primary objective of the study was to compare the mean lameness ratios between groups as a means of assessing changes in vertical force at defined times PAI. A ratio of 1 indicates the absence of pain and/or complete recovery. Conversely, a ratio of 0 indicates acute pain that prevents the dog from applying any force to the plate with the affected limb. In the control group, lameness ratio (vertical force) was 0 (i.e. non weight bearing) after 3 h post-acute pain induction, but not at 1.5 h. This can explain why the statistical comparison with firocoxib treated dogs did not show effect at 1.5 h in both experiments, because the pain was not yet installed in the control group. Looking at the control group, the model induced acute pain from 3 to 7 h after urate crystal injections, in accordance with published data [5]. In the firocoxib group the mean lameness ratios remained positive throughout both experiments, with minimum values of 0.80 at 5 h PAI in Experiment 1 and 0.36 at 3 h in Experiment 2. In the latter experiment, at 10 h PAI the lameness ratio in the firocoxib group was 0.91, with a minimum lameness ratio of 0.66, indicating that even if not always complete, analgesic efficacy was maintained through the 24-h period following treatment.

In the grapiprant group, during the two experiments, no significant difference in pain control was observed compared to the control group.

Both molecules are indicated for a daily administration, to provide anti-inflammatory and analgesic activity during 24 h [12, 13]. Elimination half-life of firocoxib is 7.59 (+/− 1.53 h), when it is 4.6 to 5.67 h for grapiprant. The Tmax is obtained in 1–3 h for both drugs [12, 13]. We can assume that the analgesic activity correlates with the plasma concentration of these molecules, which are both highly bounds to plasma proteins. It means that a decrease in efficacy could be expected in experiment 2 compared to experiment 1. It seems to be the case by comparing the firocoxib efficacy results, even if no direct comparison is possible between the two experiments which were not conducted at the same time, but at 26 days interval. The urate crystal injections were not performed in the same joint for the two experiments, which also renders direct comparisons impossible.

Based on the mode of action, which could be more progressive for grapiprant than for firocoxib, this model of acute pain control could not be sufficient to show pain and anti-inflammatory control under chronic conditions like osteoarthrosis [14].

The high level of analgesia following firocoxib treatment found in this study aligns with earlier studies [5, 8, 11]. Other force plate studies in dogs using a similar urate crystal model of induced synovitis showed that firocoxib produced substantial improvements in lameness, and three studies found that lameness in firocoxib treated groups was significantly less than in carprofen or robenacoxib treated dogs [5, 7, 10]. Force plate assessments of analgesia following tibial plateau levelling osteotomy found that firocoxib provided analgesia that was superior to robenacoxib, tramadol, and hydrocodone in three separate studies [15, 16]. The findings of force plate studies align with three field studies in which dog owner evaluations found significant improvements for firocoxib over etodolac, carprofen and deracoxib [2, 17, 18].

Grapiprant is a newly introduced drug, and so reports of testing under laboratory and field conditions have been limited to studies that are required for regulatory approvals. To date, the only report of grapiprant efficacy has been of a field study in which it was demonstrated to be superior to placebo [14]. The study we report is therefore the first in which grapiprant-induced analgesia has been assessed using a force plate analysis, and the first in which grapiprant has been compared with another NSAID. Given that grapiprant was shown to be effective in a field study, the findings of no significant difference between the grapiprant and control groups at any point in the study are surprising, particularly as for some dogs in the grapiprant group there was no evidence of any analgesic effect. This may be due to the severity of acute pain that this model generates, demonstrated by the control dogs which were unable to bear any weight on their arthritic limb.

The individual variability between dogs was important, especially in the treated groups, which is related to the individual behaviour and feeling facing pain. It thus decreases the power of statistical analysis when groups are formed by only 6 dogs. Nevertheless, for ethical reasons and practical capacities, it is difficult to increase this number, which was anyhow sufficient to show significant results in the firocoxib group.

Thus a study with a much larger number of dogs, a model that produces less intense pain, or after several daily administrations might allow achieving statistical significance between grapiprant-treated and untreated controls. Nonetheless, under the conditions of this study in which the groups were treated identically, pain control in firocoxib-treated dogs was significantly greater than that in grapiprant-treated dogs.

## Conclusion

Treatment with firocoxib prior to induction of acute arthritis in dogs resulted in a high level of analgesia from the first post-treatment assessment at 3.5 h through 24 h post-treatment. The analgesia provided by firocoxib was consistently superior to that provided by grapiprant at any point.

## Methods

The objective of the study was to measure the analgesic activity, potency and persistence of analgesia over 24 h, of a single oral dose of firocoxib (Previcox®; Boehringer Ingelheim) and grapiprant (Galliprant®, Elanco Animal Health) in an induced synovitis model of acute arthritis and pain in dogs.

Two separate experiments were conducted at 26 days of interval. Such wash-out period allowed re-using the same dogs, without changing the groups.

### Animals

Eighteen healthy Beagle dogs with no history of lameness or gait abnormality, aged from 12 to 41.5 months and weighing from 8.7 to 13.5 kg, were selected for the study from the facility kennel of the research centre (Avogadro LS, France). Dogs were acclimatized for three weeks prior to beginning the study and had been trained to lead-walk at constant speed across a force plate set into a path 50 cm wide and approximately 5 m long, maintaining a similar speed on each walk. Dogs were excluded from the study if they had received any anti-inflammatory or opiate drugs during the four weeks preceding the study.

The dogs were ranged by weight and randomly allocated in the three groups by blocks of three.

At the end of the study, all dogs were re-housed in their facility kennel.

### Treatment

Group 1 dogs were untreated controls; Group 2 dogs were treated orally with firocoxib (Previcox®, Boehringer Ingelheim) at a dose rate of 5.7 to 8.5 mg/kg; Group 3 dogs received grapiprant (Galliprant®, Elanco Animal Health) administered orally at a dose rate of 1.5 to 2.9 mg/kg following approved labels in Europe [12, 13]. The appropriate tablet(s) was placed in the back of the throat and water (5 mL) was administered via a syringe to ensure that the product was correctly swallowed. A single treatment with each product was administered for each of the two experiments, at 26 days interval. Body weights used to determine the dose of each product were taken 24 h prior to each treatment. Products were administered 2 and 14 h prior to arthritis induction in Experiments 1 and 2, respectively. Dogs were fasted for at least 12 h before treatments and food was offered from four hours post-dosing.

### Urate crystal model and determining analgesic efficacy

To induce transient osteoarthritis, dogs were anaesthetized with propofol (dose rate 6.5 mg/kg), and an intra-articular injection of 1 mL of a sodium urate crystal suspension was made into the femorotibial joint using a 30 mm by 1 mm sterile needle (Experiment 1 – right joint; Experiment 2 – left joint). All dogs were fully awake by the time of assessments (beginning 1.5 h after anaesthesia).

Such injection was shown to induce acute pain with 1.5 to 3 h, with pan remaining for 7–10 h [5, 7, 8]. All assessments were conducted at 1.5, 3, 5, 7 and 10 h post-injection in order to follow the installment of pain in untreated control dogs and its control in treated dogs.

The treatments administered 2 h before urate crystal injections in experiment 1 allowed to assess pain control at 3.5, 5, 7, 9 and 12 h post treatment.

The treatments administered 14 h before urate crystal injections in experiment 2 allowed to assess pain control at 15.5, 17, 19, 21 and 24 h post treatment.

The analgesic efficacy of each treatment was assessed on the basis of the vertical force applied by each dog’s hind limb, measured with a force plate (SATEL-Patrick Savet, Blagnac, France) connected to a computer equipped with a digital analogical acquisition card and signal processing software (Satel Véto, ENV Toulouse, France). For each assessment a dog had to be repeatedly walked cross the force plate until three interpretable hind-limb values were obtained. A video camera was used to monitor each dog’s progress across the plate. A value was considered interpretable when there was only one leg on the scale which recorded the vertical force, and when braking and propulsion phases could be differentiated on the computer output produced by the dog crossing the plate. The vertical force values were obtained at a sampling frequency of 150 Hz as each limb was placed on the force plate. The ratio between the force applied after treatment and the mean baseline force of the same hind limb measured two days before arthritis induction (the “lameness ratio” = “vertical force ratio”), was the primary variable chosen to assess pain control. Thus, if the lameness ratio for any dog approached 1, that dog was less lame because it was approaching its baseline (1 = 100% of pre-lameness induction vertical force). If severe lameness with no weight bearing was observed during the walking phase, the vertical force could not be measured and the lameness ratio was considered equal to zero.

As a second objective, visual lameness (VL) scores, from 0 to 5, were calculated for each dog, on each experiment and at each time point. The VL scores were recorded for each dog whilst standing and whilst walking (the dogs were walked for approximately one minute before scoring). The combined VL score used for statistical analysis was the sum of the standing and walking phase scores (Table 3).

**Table 3:** Scoring methodology for visual lameness observations

	Score
Observation whilst standing	
Full weight bearing (touching of the 4 digital pads on the ground)	0
Partial weight bearing (touching of 3 or less digital pads on the ground)	1
No weight bearing to toe touching	2
Observation whilst walking	
Full weight bearing, no lameness	0
Slight lameness (including intermittent) with partial weight bearing (75%): lameness barely perceptible throughout almost the whole observation period	1
Moderate lameness with partial weight bearing (□ 50%): the animal rests the limb on the ground slightly	2
Severe lameness with no weight bearing: the animal uses its limb but it does not put its weight on the limb and/or avoid putting the limb on the ground	3

Measurements (VL scores and force plate values) were collected two days before arthritis induction (Time 0) and 1.5, 3, 5, 7, and 10 h post-induction, corresponding to 2.5, 5, 7, 9 and 12 h post-treatment in Experiment 1, and to 15.5, 17, 19, 21 and 24 h post-treatment in Experiment 2. All measurements were recorded by two trained investigators who were blinded to treatments.

### Statistical analysis

Statistical analyses were performed using SAS© software version 9.4 (SAS Institute Inc., Cary, NC, USA) under Microsoft Windows© OS. A two-way analysis of the variance (ANOVA) with repeated measurements on Time was performed to analyse the Lameness ratios with the factors: Group (Control, Firocoxib, Grapiprant) and Time (1.5 h, 3 h, 5 h, 7 h, 10 h) as fixed effects; Group*Time as an interaction; and Dog ID (Group) as random effect for repeated measurement over Time. Estimated least squares means and standard error of the mean were calculated. The Visual Lameness Score parameters data were analysed through ANOVA Test involving the two fixed effects Group and Time. A significance level of α = 0.05 tested statistical significance of fixed effects.

## Data Availability

The signed final report and raw data generated and/or analysed during the current study are not publicly available due to their proprietary nature but are available from the corresponding author on reasonable request. Final report and Raw data are stored at Boehringer-Ingelheim Lyon, France.

## Abbreviations

COX: cyclooxygenase
NSAID: Non-steroidal anti-inflammatory drug
PAI: post-arthritis induction
VL: Visual lameness
