# Correction to: Assessment of the efficacy of firocoxib (Previcox®) and grapiprant (Galliprant®) in an induced model of acute arthritis in dogs

**DOI:** 10.1186/s12917-019-2116-1

**Published:** 2019-10-17

**Authors:** Andrea García de Salazar Alcalá, Lucile Gioda, Alia Dehman, Frederic Beugnet

**Affiliations:** 1 Avogadro LS, Parc de Génibrat, 31470, Fontenilles, France; 2 Hyphen-stat, 195, route d’Espagne BP13669, 31036 Toulouse Cedex 1, France; 3 Boehringer Ingelheim Animal Health, 29, avenue Tony Garnier, 69007 Lyon, France

**Correction to: BMC Vet Res (2019) 15:309**

**https://doi.org/10.1186/s12917-019-2052-0**

The original article [1] contains two mistaken instances of the molecule ‘fipronil’ in the results. These should instead have stated ‘firocoxib’.
